# The association of endodontic prognostic factors with the presence of periapical lesion, its volume, and bone characteristics in endodontically treated molars: a cross-sectional study

**DOI:** 10.1186/s12903-023-03818-x

**Published:** 2024-01-05

**Authors:** Nazanin Zargar, Kamyar Khosravi, Saeede Zadsirjan, Yaser Safi, Mohammadreza Vatankhah, Alireza Akbarzadeh Baghban, Fatemeh Aghajani Varzaneh

**Affiliations:** 1 Department of Endodontics, School of Dentistry, Shahid Beheshti University of Medical Sciences, Tehran, Iran; 2 Iranian Center for Endodontic Research, Research Institute of Dental Sciences, School of Dentistry, Shahid Beheshti University of Medical Sciences, Tehran, Iran; 3 Department of Oral and Maxillofacial Radiology, School of Dentistry, Shahid Beheshti University of Medical Sciences, Tehran, Iran; 4 School of Dentistry, Research Institute of Dental Sciences, Shahid Beheshti University of Medical Sciences, Tehran, Iran; 5 Proteomics Research Center, Department of Biostatistics, School of Allied Medical Sciences, Shahid Beheshti University of Medical Sciences, Tehran, Iran; 6 Private Dental Practice, Isfahan, Iran

**Keywords:** Cone-beam computed tomography, Cross-sectional study, Lesion volume, Periapical periodontitis, Prognostic factor, Root Canal Therapy

## Abstract

**Background:**

This study intended to evaluate the association between several endodontic prognostic factors with the presence of periapical lesions (PLs), their volume, and bone characteristics including cortical bone destruction (CBD) and buccal plate bone height (BPBH) in root-filled molar teeth using cone-beam computed tomography.

**Methods:**

A collection of 143 scans of endodontically treated maxillary/mandibular first or second molars recorded over 8 years, were obtained from a specialized radiology center. Data on prognostic factors including tooth number, gender, jaw type, the status and number of missed canals, obturation length, restoration type, presence of a separated instrument, presence of a post or screw in the canals, and presence of perforation were collected. The assessed outcomes included PL presence, PL volume, CBD, and BPBH. The association between prognostic factors and outcomes was evaluated using multiple logistic regression models with adjusted covariates and multifactorial ANOVA at a significance level of 0.05.

**Results:**

A total of 282 molars from 82 women and 50 men with a mean age of 40.6 ± 12.27 were included. Among those, 139 teeth presented PL with a mean volume of 18.68 mm^3^. CBD was prevalent in 137 teeth and the mean BPBH appeared to be 9.45 mm. The presence of a missed canal (OR = 10.022, *P* < .05), underfilled canal (OR = 3.725, *P* < .05), overfilled canal (OR = 15.859, *P* = .018), and perforation (OR = 15.261, *P* = .013) was significantly associated with PLs. None of the prognostic factors could considerably contribute to the CBD (*P* > .05). The presence of a missed canal was positively associated with the PL volume (*P* < .05). Similarly, missed canals (*P* < .05), perforation (*P* < .05), and separated instruments (*P* = .004) were associated with a significantly reduced BPBH.

**Conclusions:**

Overfillings, perforations, missed canals, and underfillings were identified as remarkable predictors of PL, arranged in descending order of their respective impact. The only factor capable of significantly increasing the PL volume was the missed canal. In brief, obturation length errors, perforations, missed canals, and separated instruments were robustly correlated with endodontic failure, which highlights the importance of mitigating the potential for errors by following the fundamentals of endodontics.

## Background

Endodontic therapy aims to disinfect the root canals thoroughly, restoring function back to teeth that may not be preserved otherwise. Microorganisms that reside on the canal walls following inadequate treatment are the fundamental causes of periapical lesions (PLs) in the alveolar bone [1]. PLs are considerably more prevalent in teeth with previous endodontic treatment than in untreated teeth, with the literature reporting a 41% compared to 3.5% frequency, respectively [2]. Several risk factors have been attributed to the prevalence of periapical disease in root-filled teeth, i.e., inhomogeneity of filling material [3], underfilled root canals [3–5], overfilled root canals [3, 5], presence of missed canals [6–8], poor coronal sealing [9], type of the restorative material [10] and procedural accidents [11].

PL size independently serves as a strong predictor of endodontic treatment success [12, 13], affecting the periapical tissue healing following non-surgical endodontic treatments [14]. Lesions less than 5 mm in diameter respond better to primary/secondary endodontic treatment than larger lesions [14–16]. Correspondingly, PL volume has been associated with endodontic outcomes so teeth with lesions larger than 50–60 mm^3^ have a significantly lower success rate following endodontic surgery [17, 18]. Moreover, the PL volume and the extent of bone destruction are positively linked to the bacterial count and levels of endotoxin found within the canal [19].

To evaluate the PLs, both two-dimensional (2D) and three-dimensional (3D) imaging techniques have been frequently used. However, given the shortcomings of the conventional radiographs, i.e., distortion and superimposition of structures, the presence of PLs might be obscured [20]. A thick cortical plate might hamper the detection of PL compared to a thinner bony plate [21]. Moreover, trabecular bone density is another factor that might negatively affect the recognition of the PL in 2D radiographs [22]. PL can be diagnosed via conventional techniques when the disease is progressing more invasively [23], making it more difficult to evaluate the subtle changes in the size/volume of the PLs [24]. Overall, cone-beam computed tomography (CBCT) scans have been demonstrated to detect PLs with higher sensitivity and accuracy than conventional radiographs [20, 21]. They also have facilitated the detection of normal anatomical landmarks, pathologies, and iatrogenic procedures with a higher resolution [8, 20].

While numerous cross-sectional studies have retrospectively evidenced the correlation between different endodontic prognostic factors and the prevalence of PLs using CBCT imaging, research towards the association of those factors and the volume of PLs is lacking in the literature. Nevertheless, preoperative PL size and volume have been revealed to significantly affect the periradicular microsurgery outcome [18]. To the best of our knowledge, our study is among the first to explore this particular association. This information can serve as a guiding tool for clinicians to discern the factors influencing PL formation. It helps identify which factors exhibit a stronger propensity to result in larger PLs. Consequently, clinicians can make more informed treatment decisions, thereby refining the prognosis of endodontically treated teeth. By advancing their knowledge and practical expertise in those areas, clinicians can potentially enhance overall patient outcomes by contributing to improved management strategies for patients.

The present study primarily aimed to evaluate the association between several endodontic prognostic factors including the presence/number of missed canals, obturation length, separated instrument prevalence, perforation, and restoration type, with the presence of PL and its volume around the roots of mandibular and maxillary pretreated molars using CBCT scans. As the secondary objective, the associations between those prognostic factors with cortical bone destruction (CBD), and buccal plate bone height (BPBH) were assessed. The null hypothesis indicates no association between the prognostic factors and the presence/volume of the PLs around the roots.

## Methods

The protocol of this study was approved by the ethics committee of Shahid Beheshti University of Medical Sciences (IR.SBMU.DRC.REC.1400.133). Contents of this research were reported in accordance with the STROBE statement for cross-sectional studies.

### Sample size calculation

Considering the type one error at *α* = 0.05, the type two error at *β* = 0.1 (90% power), and an estimated effect size of *r* = .2 as an amount in between low (*r* = .1) and moderate (*r* = .3) effect sizes, the sample size was calculated to be 265. However, 312 samples were initially entered to compensate for the potential exclusions. The formula provided below was used for sample size calculation:

$$N =( \frac{{Z}_{1-\alpha /2}+{Z}_{1-\beta }}{\frac{1}{2} {\text{log}}_{n}\left(\frac{1+r}{1-r}\right)})^{2} + 3$$

### Image acquisition and assessment

A total of 143 CBCT scans of 312 endodontically treated maxillary/mandibular first or second molars of 132 patients, recorded between 2013 and 2020, were acquired from a specialized oral and maxillofacial radiology center. Patients’ information was kept confidential throughout the study. All scans were obtained by SCANORA 3D (Soredex, Tuusula, Finland) with a voxel size of 200 μm and a field of view (FOV) of 7.5 × 10 cm, with 90 kVp and 6 mAs. Images were observed using OnDemand3D (Cybermed, Seoul, South Korea) on a 14-inch, 1920 × 1080 pixels resolution, 60 Hz monitor. Contrast and brightness were manually adjusted using the windowing tool to achieve the optimal image quality. To ensure proper orientation, vertical and horizontal lines were aligned parallel to the long axis of the roots being examined, and the inspection was performed in axial, sagittal, and coronal planes as appropriate.

Endodontically treated teeth were assessed by a preoperatively calibrated senior dental student, under the direct guidance of an experienced endodontist and an oral and maxillofacial radiologist, for detection of the prognostic factors relevant to this study. Exclusion criteria were as follows: teeth with untreated roots, low-quality scans, scans with varying voltages, presence of artifacts, history of previous endodontic surgery, presence of vertical root fracture, presence of external root resorption, presence of furcal lesions, strip perforation (only if it led to a furcal lesion), PLs with apicomarginal communication, and teeth with PL extending to roots of adjacent teeth.

### Data collection

**Fig. 1:**
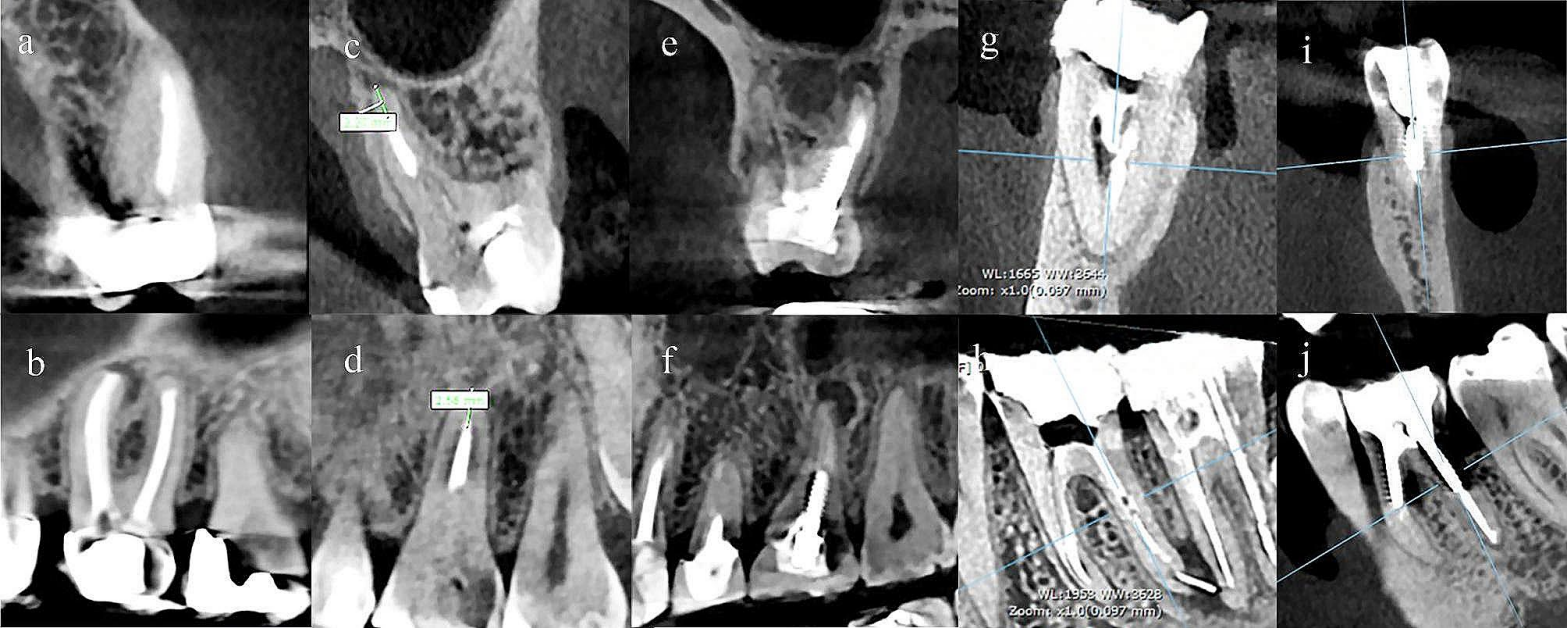
(**a, c, e, g**, and **i**): Coronal view of the teeth, (**b, d, f, h**, and **j**): Sagittal view of the teeth. (**a, b**): Upper left first molar with missed MB2 canal, PL, and a PFM crown; (**c, d**): Upper left first molar with a underfilled palatal canal; (**e, f**): Upper right first molar with a screw in the palatal canal, PL, and composite restoration; (**g, h**): Lower right first molar with separated instrument in the distal canal and PL; (**i, j**); Lower left first molar with furcal lesion (excluded from the study)

The data from independent variables recorded by the examiner were as follows: tooth number, gender, jaw type, the status of missed canals and their quantity, obturation material length, type of restoration, presence of a separated instrument, presence of a post/screw in the canals, presence of perforation (Fig. 1). Length of obturation was classified as appropriate if the ending was at a distance of 0–2 mm from the apex, underfilled when it was shorter than 2 mm from the apex, and overfilled if the material extruded the apex [3].

**Fig. 2:**
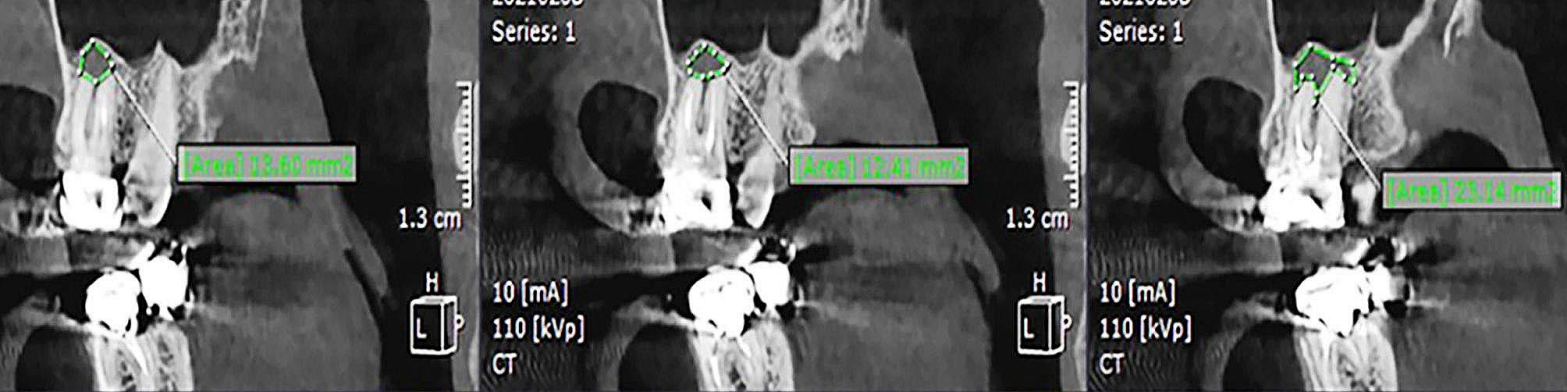
Determining the surface area of the PL in three consecutive slices using the area tool

Data on the four dependent variables were recorded: presence of PL, PL volume, presence of CBD, and BPBH. PL was identified as absent when intact periapical bone structures could be detected (diameter of the periapical radiolucency under 0.5 mm), and otherwise as present [25]. In order to evaluate PL volume, the tooth was viewed in the sagittal plane with one-millimeter interslice intervals. The surface area of the PL in each slice was measured using the area tool in the software (Fig. 2). Considering the PL as an asymmetrical 3D object, its volume was measured using the formula presented below, where h represents the interslice interval, and S denotes the surface area of partial frustum bases. The accuracy of this measurement method has been proven in several publications [18, 26, 27].

$$V= \frac{h\times (S1+ \sqrt{S1 \times S2}+S2)}{3} + \frac{h\times (S2+ \sqrt{S2 \times S3}+S3)}{3} + \cdots$$

CBD was observed from the coronal plane slices, showing the buccolingual bone surrounding the roots. BPBH was defined using the ruler tool to measure the apicocoronal height of the bone covering the roots.

### Statistical analysis

Datasets were entered into the SPSS software version 26.0 (IBM, Armonk, NY, USA). To analyze the association between prognostic factors and the presence of PL or CBD, a multiple logistic regression model was employed to adjust for covariates. To investigate the association between the PL volume or BPBH with the prognostic factors, multifactorial ANOVA was conducted. *P* < .05 was considered as statistically significant.

Intraexaminer reliability was determined using Cohen’s Kappa test. A single examiner (F.A.) inspected 40 CBCT scans, chosen randomly from the study population, to detect measures on the PL status, obturation length, and CBD status. A re-examination was performed 21 days later. The results were compared and the Kappa statistic was calculated, considering values over 0.6 as substantial to perfect agreement [28].

## Results

Kappa scores of 0.95, 0.84, and 0.89 for PL status, obturation length, and CBD status were achieved, respectively, suggesting almost perfect intraobserver reliability.

Totally, 282 molars from 82 women (103 upper molars, 78 lower molars) and 50 men (72 upper molars, 29 lower molars) with a mean age of 40.6 ± 12.27 were included in the study. Thirty teeth were excluded due to strip perforation leading to a furcal PL. The descriptive characteristics of the included teeth are presented in Table 1. In total, 139 teeth presented with a PL with a mean volume of 18.68 mm^3^. CBD was prevalent in 137 teeth and the mean BPBH appeared to be 9.45 mm. A statistically significant association between the tooth type and the PL frequency (*P* = .021) and its volume (*P* = .006) was observed with both factors being greater in maxillary molars (Table 2). BPBH was significantly greater in the absence of PL (*P* < .05), while no statistical association (*P* = .407) was found between the PL status and CBD (Table 3).

**Table 1:** Descriptive information of the study population

Independent variable	Subgroup	Sample size, n (%)	PL status		Mean PL volume (mm^3^)	Cortical bone destruction status		Mean buccal plate bone height (mm)
			Present, n (%)	Absent, n (%)		Destructed, n (%)	Intact, n (%)	
Gender	Female	181 (64.2%)	86 (47.5%)	95 (52.5%)	18.24	90 (%)	91 (%)	9.48
	Male	101 (35.8%)	53 (52.5%)	48 (47.5%)	19.48	47 (%)	54 (%)	9.39
Jaw type	Upper	175 (62.1%)	100 (57.1%)	75 (42.9%)	23	88 (50.3%)	87 (49.7%)	9.25
	Lower	107 (37.9%)	39 (36.4%)	68 (63.6%)	11.62	49 (45.8%)	58 (54.2%)	9.77
Missed canal	Present	142 (50.4%)	100 (70.4%)	42 (29.6%)	29.4	65 (45.8%)	77 (54.2%)	8.99
	Absent	140 (49.6%)	39 (27.8%)	101 (72.2%)	7.73	72 (51.4%)	68 (48.6%)	9.91
Number of missed canals	0	140 (49.6%)	39 (27.8%)	101 (72.2%)	7.73	72 (51.4%)	68 (48.6%)	9.91
	1	132 (46.8%)	91 (69%)	41 (31%)	26.55	57 (43.2%)	75 (56.8%)	9.06
	2	8 (2.8%)	7 (87.5%)	1 (12.5%)	57.01	6 (75%)	2 (25%)	8.85
	3	2 (0.8%)	2 (100%)	0	112.71	2 (100%)	0	5.38
Obturation length	Appropriate	209 (74.2%)	88 (42.1%)	121 (57.9%)	16.86	99 (47.4%)	110 (52.6%)	9.48
	Underfilled	68 (24.1%)	47 (69.1%)	21 (30.8%)	23.68	35 (51.5%)	33 (48.5%)	9.39
	Overfilled	5 (1.7%)	4 (80%)	1 (20%)	26.88	3 (60%)	2 (40%)	8.85
Restoration	Amalgam	131 (46.4%)	62 (47.3%)	69 (52.7%)	17.13	63 (48.1%)	68 (51.9%)	9.59
	Composite	23 (8.1%)	13 (56.5%)	10 (43.5%)	35.34	9 (39.1%)	14 (60.9%)	9.29
	PFM crown	126 (44.6%)	63 (50%)	63 (50%)	17.67	63 (50%)	63 (50%)	9.33
	Extensive caries	2 (0.7%)	1 (50%)	1 (50%)	1.99	1 (50%)	1 (50%)	8.75
Separated instrument	Present	3 (1%)	3 (100%)	0	44.03	3 (100%)	0	6.21
	Absent	279 (99%)	137 (49.1%)	142 (51.9%)	18.41	134 (48%)	145 (52%)	9.48
Post	Present	78 (27.6%)	37 (47.4%)	41 (52.6%)	18.14	37 (47.4%)	41 (52.6%)	9.40
	Absent	204 (72.3%)	104 (51%)	100 (49%)	18.89	100 (49%)	104 (51%)	9.47
Perforation	Present	11 (3.9%)	10 (90.9%)	1 (9.1%)	28.29	8 (72.7%)	3 (27.3%)	7.49
	Absent	271 (96.1%)	129 (47.6%)	142 (52.4%)	18.29	129 (47.6%)	142 (52.4%)	9.53
Total	-	282 (100%)	139 (49.3%)	143 (50.7%)	18.68	137 (48.5%)	145 (51.5%)	9.45

PL: periapical lesion

**Table 2:** The association between tooth type and two dependent variables

Tooth type	PL status		*P*	Mean PL volume (mm^3^)	*p*
	Present, n (%)	Absent, n (%)	0.021*		0.006*
Maxillary first molar	74 (61.1%)	47 (38.9%)		24.23	
Mandibular first molar	25 (43.1%)	33 (56.9%)		13.69	
Maxillary second molar	25 (47.1%)	28 (52.9%)		19.95	
Mandibular second molar	15 (30%)	35 (70%)		9.68	

PL: periapical lesion, *: statistically significant level. Chi-square and independent samples T-test were employed to reveal the association between the tooth type and the PL prevalence, and between the tooth type and the PL volume, respectively

**Table 3:** The association between PL status and two dependent variables

PL status	Sample size (n)	Cortical bone destruction status (n)		*P*	Mean buccal plate bone height (mm)	*P*
Present	139	Yes	64	0.407	8.60	< 0.001*
		No	75		8.76	
Absent	143	Yes	73		10.06	
		No	70		10.33	

PL: periapical lesion, *: statistically significant level. Chi-square and independent samples T-test were employed to reveal the association between the PL prevalence and cortical bone destruction, and between the PL prevalence and mean buccal plate bone height, respectively

Considering the prognostic factors, the prevalence of missed canals (*P* < .05), underfilled canals (*P* < .05), overfilled canals (*P* = .018), and perforations (*P* = .013) could significantly affect the presence of PLs (Table 4). An overfilled canal showed a 15.8-fold greater likelihood of accompanying a PL compared to an underfilled canal with an odds ratio (OR) of 3.7. Moreover, perforation (OR = 15.2) and missed canals (OR = 10) were significant predictors for PLs to accompany root-filled molars. Regarding CBD, none of the prognostic factors could considerably contribute to its prevalence (*P* > .05).

**Table 4:** Multiple logistic regression for the association of the independent variables and two dependent variables

	Presence of the PL		Cortical bone destruction	
	**Adjusted OR (95% CI)**	***P***	**Adjusted OR (95% CI)**	***P***
Gender	0.761 (0.424–1.366)	0.360	1.200 (0.723–1.990)	0.480
Jaw type	1.427 (0.685–2.971)	0.342	0.636 (0.346–1.169)	0.145
Missed canal	10.022 (4.877–20.595)	< 0.001*	0.639 (0.356–1.146)	0.133
Obturation length^ǂ^	-	< 0.001*	-	0.762
Underfilled canal	3.725 (1.875–7.398)	< 0.001*	1.083 (0.617-1.900)	0.782
Overfilled canal	15.859 (1.611-156.137)	0.018*	1.962 (0.296–12.987)	0.484
Restoration type^ǂ^	-	0.764	-	0.546
Composite restoration	1.472 (0.516–4.195)	0.470	0.716 (0.283–1.809)	0.480
PFM crown	1.013 (0.546–1.880)	0.968	1.195 (0.708–2.017)	0.505
Perforation	15.216 (1.756-131.831)	0.013*	2.822 (0.724–10.994)	0.135
Post	0.860 (0.440–1.678)	0.658	0.875 (0.496–1.545)	0.646

PFM: porcelain fused to metal, PL: periapical lesion, OR: odds ratio, CI: confidence interval; *: statistically significant level; ^ǂ^: reference category; separated instrument domain was disregarded due to low sample size

According to Table 5, the presence of a missed canal was positively associated with the PL volume (*P* < .05) and negatively with the BPBH (*P* < .05). Correspondingly, root-filled molars with perforation (*P* < .05) or separated instrument (*P* = .004) could significantly reduce the BPBH.

**Table 5:** Multifactorial ANOVA results for the association of the independent variables and two dependent variables

	PL volume		Buccal plate bone height	
	**F**	***P***	**F**	***P***
Gender	0.042	0.838	0.245	0.621
Jaw type	0.017	0.897	0.012	0.914
Missed canal	15.124	< 0.001*	13.522	< 0.001*
Obturation length	0.817	0.443	0.201	0.818
Restoration type	2.318	0.100	0.563	0.570
Perforation	0.923	0.338	15.145	< 0.001*
Separated instrument	0.671	0.413	8.474	0.004*
Post	0.211	0.647	0.107	0.744

PL: periapical lesion, F-statistic: the mean square for the factor divided by the residual mean square, *: statistically significant level

Among the upper first and second molars, the second mesiobuccal (MB2) canal was the most commonly missed, occurring in 123 teeth, followed by a missed distobuccal canal appearing in 6 molars. In the case of lower first and second molars, 9 teeth were identified to have a missed mesiolingual (ML) canal as the most frequently missed canal.

## Discussion

The current retrospective cross-sectional study investigated the association between endodontic prognostic factors and four dependent variables including PL presence, its size, CBD, and BPBH, on CBCT scans. Our findings revealed that missed canals, perforation, underfilled, and overfilled canals were significantly correlated with the presence of PL. Specifically, missed canals emerged as the primary factor affecting the PL volume. Therefore, the null hypothesis was rejected. In addition to the missed canal, perforation and separated instrument could significantly diminish BPBH. On the other hand, none of the factors mentioned could exert a statistically considerable effect on CBD.

### Radiologic consideration

PL is a chronic manifestation of the activity of bacteria in the canal system of teeth with/without previous endodontic treatment. On a global scale, 52% of the adult population has been identified with PL in at least one of their teeth, reportedly [29]. Given the high prevalence and asymptomatic nature of PLs, their early detection via radiography remains of the utmost importance for their management. CBCT-based studies have commonly reported a higher prevalence of PL in their population compared to other studies with periapical/panoramic assessments [29, 30]. The lower detection rate of conventional radiographs might be attributed to the fact that a PL becomes discernible only when a 30-50% loss of bony mineral content has occurred [23]. Consequently, we used CBCT scans to detect the potential association of the prognostic factors and dependent variables in this paper.

Given that endodontic procedures typically target specific areas of interest, the utilization of limited FOV in CBCTs proves to be a suitable method for treatment planning and surgical strategy development when contrasted with broader FOVs. A restricted FOV not only reduces the radiation exposure to the patient but also yields higher-resolution scans, thereby facilitating a more straightforward interpretation [13]. Nonetheless, it is recommended to prioritize the use of intraoral radiographs as the preferred imaging method in assessing endodontic patients and to reserve the CBCT scans for potential morphological complexities and dental anomalies [13, 31].

### PL status

The overall prevalence of PL in this study was found to be 49.3% in endodontically treated teeth, which was in line with several previous papers [4, 30, 32–35]. However, there are other publications in the literature reporting less [3, 36, 37] or more [8, 38–41] prevalence regarding PLs. Discrepancies in the reported prevalence might stem from differences in sample size and the radiographic techniques of choice implemented to detect PLs. Overall, endodontic treatment significantly increases the probability of PL in teeth, with an OR being reported as high as 25.17 [36].

During the present study, no significant difference was found between gender and the presence of PL (*P* = .360), confirming previous research [10, 34, 42, 43]. Nevertheless, Dutta et al. reported that PL prevalence was significant towards gender with women (OR = 37.78) having higher odds than men (OR = 17.05) [32]. On the other hand, Bürklein et al. found men to be more affected by PL in their root-filled teeth than women [3]. The heterogeneous findings could be explained by variations in the sample size and uniformity, as some studies focused exclusively on root-filled teeth, while others included untreated teeth as well.

Moreover, no statistical difference was established regarding PL prevalence in maxillary/mandibular teeth in the multivariate-adjusted model (OR = 1.427, *P* = .342). However, when performing an unadjusted chi-square analysis, PL appeared to be significantly more prevalent in the maxilla (*P* = .001), which corroborates previous studies [3, 4, 42]. This trend has been attributed to the variation in bone structure between the upper and lower jaws with the mandible exhibiting a denser osseous pattern which limits the expansion of the PL. Nevertheless, accounting for predictive variables collectively by conducting an adjusted analysis limits bias, thereby ensuring more reliable results [44].

As the most prevalent technical error in this study, 72% and 15% of maxillary and mandibular molars (50.4% of all samples) were associated with a missed canal, respectively, of which 70.4% were accompanying a PL (OR = 10.022, *P* < .05). This significant association has been addressed in several publications so far, with reported OR spanning from 2.9 [3] to 6.25 [7] in various studies. Baruwa and colleagues reported a 12% prevalence of missed canals in their study, of which 82.6% were correlated with a PL (OR = 4.4) [6]. Meirinhos et al. demonstrated a 79% prevalence of PL in root-filled teeth with untreated canals [30]. The increased odds identified in our results could be a product of our homogenous study population, exclusively involving teeth that had undergone endodontic treatment.

The prevalence of MB2, which is an additional canal in maxillary molars, can be observed in as much as 60% of cases involving the first and second maxillary molars [45]. At the root level, both first and second maxillary molars predominantly exhibited missed MB2 canals, followed by missed ML canals in the lower molars, which precisely corroborates the findings of previous authors [4, 6–8]. This finding is substantiated by considering the complexity of the anatomy and the difficult accessibility of these canals. However, Karabucak et al. found the second distal canal as the most commonly missed canal in the mandibular first molars [8]. The approximate prevalence of this canal has been reported to be 22% [46]. Additionally, a recent cross-sectional study conducted across multiple countries has revealed the presence of middle mesial canals in approximately 7% of the lower first molar teeth [47]. The presumption of a three-canal system could probably avert the clinician from further negotiating for an additional distal or mesial canal upon discovering the initial ones. These results could suggest that clinicians may allocate greater attention to MB2, ML, and second distal canals in their practice to further prevent PL formation.

Obturation length was a significant predictor for PL (*P* < .05), with overfilled canals exhibiting a more prominent effect (OR = 15.859) in increasing the chance of PL than underfilled canals (OR = 3.725). A short filling was prevalent in 24.1% of cases of which 69.1% had led to the presence of a PL. Overfillings existed in only 1.7% of cases of which 80% were accompanying a PL. In this regard, Bürklein et al. reported that 31.2% and 1% of cases were underfilled and overfilled, respectively [3]. While the likelihood of association with PL regarding overfilled canals was smaller in our study than theirs (OR = 27, *P* < .05), the results for underfilled cases in our study relatively corroborated their findings (OR = 4.37, *P* < .05). In the study by Kabak et al., 29.6% and 8.3% of root-filled teeth were underfilled and overfilled, respectively [36]. Corresponding to our results, overfilled cases (OR = 5.42, *P* < .05) were associated with odds of PL more intensely than underfilled (OR = 2.59, *P* < .05) cases. El Ouarti et al. reported that inadequately filled roots were associated with PLs with an OR of 1.93 (*P* = .004) in a Moroccan subpopulation [39], while Frisk et al. reported the OR for the same association to be 2.77 (*P* < .05) in a Swedish population [10]. On the other hand, another author reported no significant difference between overfilled canals and the ones with adequate root filling length [4].

Overfillings might provoke tissue irritation due to prior overinstrumentation. They may trigger an inflammatory response by pushing infectious residue to the periapex [48]. Furthermore, overfillings might induce foreign body reactions in the periapical region even in the absence of microorganisms. This situation could result in PLs remaining asymptomatic for extended periods [49]. Similarly, the infectious dentinal debris left in the unobturated portion of the underfilled canals could aggravate the treatment prognosis by elevating the risk of a sustained infection [48].

Successful endodontic therapy relies on homogeneous obturation of the prepared roots along with a hermetic seal of the coronally destructed tooth portion, without one outweighing the other [50]. However, quality assessment of coronal restorations in CBCT scans is subject to scattering artifacts which imposes challenges in evaluating restoration quality impeccably. Therefore, clinical examination and intraoral radiography stand out as more reliable methods for appraising the quality of coronal restorations [4].

In the current study, neither the type of restoration nor the presence of a post could significantly influence the periapical status of the root-filled teeth, which corroborates the findings of a previous study [39]. Conversely, according to Hommez et al., teeth with composite restorations demonstrated a higher association with PL than teeth restored with amalgam (*P* < .01). Meanwhile, it was noted that the use of posts had no apparent effect on the status of PL [51].

A previous study has demonstrated a significant association between crowns and PLs, while reporting no correlation toward PL status in teeth restored with plastic fillings [4]. The observed correlation has been attributed to greater structural tooth loss preceding crown placement. However, Frisk et al. have reported a significant association between plastic restorations (composite and large mixed amalgam and composite) with PL (*P* < .05) whereas they did not find a significant correlation regarding full crowns and PL status (*P* = .06) [10]. These inconsistencies could be accounted for by the retrospective nature of the research, which falls short of providing details about the condition of teeth prior to treatment. In sum, the integrity of the coronal restoration seems to hold more significance than the choice of material.

In this study, a 3.9% prevalence of perforation was noted, of which 90.9% were linked to a PL (OR = 15.216, *P* = .013). Yamaguchi and colleagues have reported perforation as the second most important cause of treatment failure in 11.8% of root-filled teeth in a Japanese population [52]. The inadvertent creation of a pathological connection between the root and its surrounding tissues could potentially result in complications severe enough to require the removal of the affected tooth. However, nonsurgical repair of perforations has been reported to display clinical success rates as notable as 70% [53].

### PL volume

The literature has covered various methods for measuring the volume of 3D objects, including the water displacement method (WDM), geometric-based models, magnetic resonance imaging, and CT-based techniques [27, 54, 55]. WDM is considered the gold standard, providing optimal precision in determining the volume of an object by dipping it in a water-filled container and measuring the overflow [56]. However, this measurement is only achievable with externally replicated PLs [55]. CBCT scans have provided comparable results to WDM in terms of accuracy [27, 54, 55]. However, precise detection of the PL borders via this method is a time-consuming and laborious procedure. Geometric-based models including partial frustum have demonstrated promising accuracy in cases where the previous two methods have been impossible to employ [27]. Therefore, we implemented a partial frustum model on CBCT scans, as discussed in the methods section, to have an accurate assessment of the PL volume.

The results suggested that the presence of missed canals was the only factor exerting a significant association with PL volume. The volume of PL was measured at 29.4 mm^3^ in the presence of missed canals and 7.73 mm^3^ in their absence (*P* < .05). Moreover, PL volume increased from 26.55 mm^3^ to 112.71 mm^3^ in teeth with one to three missed canals, respectively. Maxillary first molars had the greatest PL volume which was significantly larger than other types of teeth (*P* = .006). This larger volume could be attributed to the high prevalence of the missed MB2 canals in these teeth.

### Cortical bone destruction and buccal plate bone height

Whether these factors could potentially be linked to the outcome of endodontic surgery remains a matter of debate. While Song et al. have indicated a preoperative BPBH > 3 mm and the presence of buccal cortical plate as significant predictors for endodontic microsurgery success (*P* < .05) [57], other authors have failed to find such a relationship [18, 58]. Nonetheless, the pooled results of a recently published systematic review and meta-analysis indicate that while a BPBH > 3 mm may marginally enhance the microsurgery outcome, the observed distinction from cases with a BPBH below 3 mm did not reach statistical significance [16].

In this study, 48.5% of teeth presented with CBD, and none of the variables had a significant effect on CBD (*P* > .05). Meanwhile, the presence of the PL, missed canal, perforation, and separated instrument were negative predictors of the BPBH (*P* < .05).

### Limitations, strengths and recommendations

Cross-sectional studies present inherent shortcomings when assessing the progression of PLs. Due to the absence of information on teeth’s preoperative pulpal and periapical diagnosis, dental signs and symptoms, patients’ medical history, and the treatment date as the baseline, it is impossible to anticipate if PLs are healing or advancing. Thus, certain PLs associated with root-filled teeth may not indicate active diseases, thereby hindering the drawing of definitive conclusions regarding their association. Consequently, further rigorously controlled cohort or case-control studies are warranted. These studies should encompass all the aforementioned factors along with a comprehensive evaluation of the endodontic-periodontal relationship, to yield more generalized and valid results.

Another limitation of our study is the acquisition of CBCT scans from a single center. To enhance the results’ reliability, we recommend conducting similar research on a broader, multicenter national or international scale. Additionally, our method for measuring PL volume is relatively innovative, suggesting the need for further investigations using alternative approaches, such as specific CBCT software, to compare and improve the generalizability of results.

On the positive side, our study stands out by pioneering the PL volume measurement and exploring its correlation with endodontic prognostic factors. Moreover, we have performed an adjusted analysis to incorporate various prognostic factors, ensuring comprehensive statistical reporting of their collective impact. Comprehending this association, clinicians can precisely evaluate the treatment prognosis and establish an efficient treatment plan to enhance the patient’s well-being.

## Conclusions

Within the limitations of this study, overfillings, perforations, missed canals, and underfillings were identified as noteworthy predictors of PL, arranged in descending order of their respective impact. The sole factor capable of significantly increasing the PL volume was the missed canal. Furthermore, missed canals, perforations, and separated instruments were perceived as significant negative indicators of BPBH. In short, obturation length errors, perforations, missed canals, and separated instruments were strongly associated with endodontic failure, which underscores the importance of mitigating the potential for errors by adhering to the fundamentals of endodontics.

## Data Availability

The data pertaining to this research is accessible and can be obtained by reaching out to the corresponding author, subject to a reasonable request.

## Abbreviations

2D: Two dimensional
3D: Three dimensional
BPBH: Buccal plate bone height
CBCT: Cone-beam computed tomography
CBD: Cortical bone destruction
FOV: Field of view
OR: Odds ratio
PL: Periapical lesion
WDM: Water displacement method
